# The Future Maternity Services of Bristol

**Published:** 1970-01

**Authors:** Joseph Sluglett

**Affiliations:** General Medical Practitioner, Bristol


### Looking Ahead: The Future Maternity Services of Bristol

**Joseph Sluglett, O.B.E., M.D.**
General Medical Practitioner, Bristol

The proposals described in this paper are concerned mainly with the role of the general practitioner but quite obviously, they must be considered within the framework of the maternity services as a whole. The G.P. has nevertheless a most important part to play since usually, he is the first person to be consulted by the expectant mother.

It must be emphasised that in Bristol there is a very happy relationship between the three branches of the tripartite organisation, District Midwives, G.P.s and Consultant Obstetricians. There is no shortage of maternity beds and it is now possible for any patient who so wishes and who satisfies the accepted criteria, to be delivered in Wendover, St. Brenda's, or the Walker Dunbar maternity hospitals which have a total of 74 beds. There is, in addition, the short stay unit at Southmead Hospital temporarily closed for extensive rewiring but for which alternative accommodation is being provided at Bristol Maternity Hospital. Our records can bear comparison with the best in the country, yet, as shown by the latest report on Confidential Enquiries into Maternal Deaths (1969), there is no room for complacency. We must be continually searching for ways to improve our services.

Any plans for the future must take into account a number of factors of which the most important is the increasing demand by mothers for institutional delivery whether in specialist or GP maternity hospitals. In 1967 only 16% of live births took place at home while by 1968 this had dropped to 11% and it is reasonable to assume that this figure will be further reduced in the future. This is a trend which many feel should be encouraged, there is no place for domiciliary delivery in a well equipped town like Bristol.

Another factor is the increasing number of G.P.s who, for one reason or another no longer wish to undertake midwifery as part of their National Health Service and this naturally throws more work on to those of their colleagues who enjoy it. There is some reason to suppose that many more G.P.s would be willing to give up their midwifery if satisfactory arrangements could be made. On the other hand there are some G.P.s who would like to do more but find it impossible because of the nature of their practices.

There are two other factors to be considered in the assessments of future needs. The first of these is the fall in the birth rate from 7309 in 1967 to 6988 in 1968. This fall will very likely be accelerated by the increasing use of the "pill", the "loop" and other contraceptive methods as well as the increasing demand for sterilisation of grand multipara and terminations of pregnancy under the Abortion Act. Secondly, there is the high cost of hospital care though this may be modified by more extensive application of the principle of early home discharge in all suitable cases.

Much more important than all this is the health and safety of the expectant mother and present thinking is that these can best be safeguarded in units specially designed and equipped for this purpose.

Until quite recently most G.P.s thought, that as far as their cases were concerned special G.P. maternity hospitals would suffice but there is increasing realisation that this is not the answer nowadays. They would feel much happier if they could conduct their deliveries in a well equipped maternity hospital where all facilities would be immediately available in case of emergency. Further, if all mothers are to benefit from the modern approach to labour as described by O'Driscoll (1969) and his colleagues in Dublin, this can only be done in a maternity hospital.

It is proposed that all deliveries in the future should take place in Bristol Maternity Hospital or Southmead Hospital and that all the other maternity hospitals be closed. The delivery would be followed, where there are no clinical or social contraindications, by immediate home discharge. This policy, first suggested in a Lancet article (Sluglett and Walker, 1956) has been successfully adopted in different parts of the country and two papers in 1967 (Arthurton and Bamford; Craig and Muirhead), showed quite convincingly that neither mother nor baby suffered any harm on this account. One or two of the G.P. hospitals may be used for the after care of mothers otherwise well who, for social reasons cannot go home, or, as at present, for antenatal care in minor complications or where the patient is simply in need of a rest from her household chores.

The general practitioner obstetricians would be on the staff of the hospital on a part-time basis, each being attached to the consultant of his choice and they must be willing to accept the responsibilities and discipline such an appointment entails. It is an important part of the scheme that there should be a strict obstetric list so as to create a group of G.P.s who are deeply interested in this subject and are willing to undertake the extra work involved. This would mean that far fewer G.P.s would do a lot more midwifery and in this way they would gain confidence, experience and awareness of the dangers which beset every expectant mother; there is no room for the occasional obstetrician. These general practitioner obstetricians would be responsible for all cases coming within the accepted criteria and attend them for antenatal care at the Local

Authority clinics and Health Centres in various parts of the city; they would be expected to be present at delivery or arrange for a deputy. There would be no distinction between G.P. and consultant cases, all mothers would come into the same unit under the same obstetric team. If any difficulty arose the G.P. would summon help in the same way as the houseman asks for the registrar or consultant.

It would be extremely difficult for any G.P. with a busy practice to do the extra work this would entail without special arrangements being made. It is therefore suggested that, as part of their contract with the maternity hospital they would have regular sessions of duty at the hospital to deal with all cases as they come in. This would not preclude them coming in to attend to a particular case but since comparatively few mothers would be their own patients this difficulty would not often arise. In any case, by reason of absence through holidays, sickness or study leave there is bound to be a fair amount of sharing of patients.

In a group practice or partnership there would be no difficulties as the work load could be organised for those occasions when the G.P.O. is absent but there is nothing to stop the singlehanded G.P. making arrangements for a colleague to look after his patients and naturally these services would be paid for.

In effect the G.P.O.s would be engaged in obstetrics for one or two days a week and in general practice for the rest of the time. This would have a great appeal for many G.P.s who enjoy this branch of medical practice. In much the same way the district midwives while remaining under the administration of the Local Health Authority would be organised as part of the obstetric team and they too would be on the staff of the hospital. They would attend the antenatal clinics with the G.P.O.s and have regular sessions in the labour wards. They would take the patient home by ambulance and with the G.P.O. be responsible for the postnatal care.

It is impossible at this stage to make any assessment of the numbers of G.P.O.s, midwives or labour wards required for such a scheme; this would only emerge after discussion with all the interested parties. It is hoped that the Regional Hospital Board will consider these proposals in the light of the building programme of the new Maternity Hospital and the extensions at Southmead.

One great advantage of the scheme is that no vast expenditure of public money is involved. As far as the hospitals are concerned what is required is the conversion of ordinary beds into labour rooms. These would not require specially expensive equipment since there would be full operating theatre facilities within immediate reach if required. The district midwives would be paid by the Local Health Authority as at present and extra staff would be available from the closure of the G.P. hospitals.

As for the G.P.O.s, they would be paid as at present from funds recovered from the Local Executive Councils through forms E.C.24 which every expectant mother signs at her first attendance at the antenatal clinic. Fees from this source which would be augmented by fees for attendance at miscarriages would provide an adequate pool to be shared out among the G.P.O.s. They could be paid on an "item of service" basis or, as is much more likely since all would be doing roughly the same amount of work, by salary. This would recompense them for the extra work and responsibilities they undertake and also enable them to make satisfactory financial arrangements with their colleagues.

It is however the expectant mother who would be the real beneficiary in such a scheme. There is no doubt that the close association of general practitioner obstetricians and midwives with the hospital staff would do much to raise and keep at a high level the standards of maternity care in Bristol.

### REFERENCES

**Arthurton M. W., and Bamford, F. N.;** Pædiatric Aspects of the Early Discharge of Maternity Patients (1967). *Brit. med. J.* **3,** 517.

**Craig, G. A., and Muirhead, J. M. B.;** Obstetric Aspects of the Early Discharge of Maternity Patients (1967). *Brit. med. J.* **3,** 520.

**O'Driscoll, K., Jackson, R. J. A., and Gallagher, J. T.;** Prevention of Prolonged Labour (1969). *Brit. med. J.* **2,** 477.

Report on Confidential Enquiries into Maternal Deaths in England and Wales, 1964-1966, Department of Health and Social Security, London H.M.S.O., 1969.

**Sluglett, J., and Walker, S.;** A General-Practitioner Maternity Unit (1956). *Lancet* **1,** 684.

